# Compartmentalizing and sculpting nanovesicles by phase-separated aqueous nanodroplets†

**DOI:** 10.1039/d2ra05855c

**Published:** 2022-11-08

**Authors:** Fatemeh Kazemi Sabet, Arash Bahrami, Amir H. Bahrami

**Affiliations:** School of Mechanical Engineering, College of Engineering, University of Tehran North Kargar St. 14399-57131 Tehran Iran; UNAM-National Nanotechnology Research Center and Institute of Materials Science & Nanotechnology, Bilkent University Ankara Turkey bahrami@unam.bilkent.edu.tr; Living Matter Physics, Max Planck Institute for Dynamics and Self-Organization 37077 Göttingen Germany

## Abstract

Phase-separated liquid droplets inside giant vesicles have been intensely studied as biomimetic model systems to understand cellular microcompartmentation and molecular crowding and sorting. On the nanoscale, however, how aqueous nanodroplets interact with and shape nanovesicles is poorly understood. We perform coarse-grained molecular simulations to explore the architecture of compartmentalized nanovesicles by phase-separated aqueous nanodroplets, and their morphological evolution under osmotic deflation. We show that phase separation of a biphasic liquid mixture can form both stable two-compartment and meta-stable multi-compartment nanovesicles. We identify morphological transitions of stable two-compartment nanovesicles between tube, sheet and cup morphologies, characterized by membrane asymmetry and phase-separation propensity between the aqueous phases. We demonstrate that the formation of local sheets and in turn cup-shaped nanovesicles is promoted by negative line tensions resulting from large separation propensities, an exclusive nanoscale phenomenon which is not expected for larger vesicles where energetic contributions of the line tensions are dominated by those of the membrane tensions. Despite their instability, we observe long-lived multi-compartment nanovesicles, such as nanotubules and branched tubules, whose prolonged lifetime is attributed to interfacial tensions and membrane asymmetry. Aqueous nanodroplets can thus form novel membrane nanostructures, crucial for cellular processes and forming cellular organelles on the nanoscale.

## Abstract 2

Internal aqueous nanodroplets form novel stable and long-lived unstable nanovesicles, exclusive to the nanoscale and crucial for membrane nanostructures.

## Graphical abstract

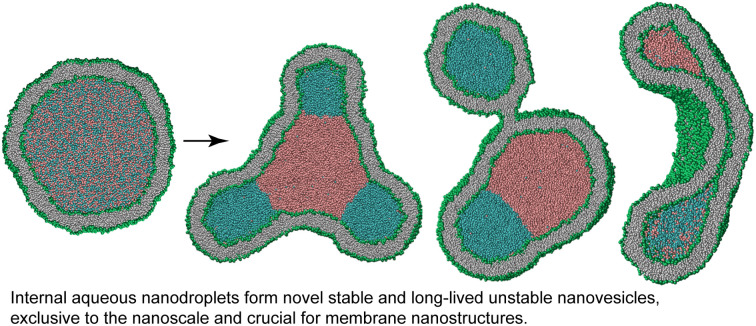

## Introduction

Lipid vesicles, loaded with aqueous droplets, serve as model systems to explore several biological processes in living cells. These processes include cellular compartmentation by phase-separated biomolecular condensates,^1–3^ vesicle budding,^4–6^ membrane tube formation,^7^ and clustering of synaptic vesicles by synapsin droplets.^8–10^ Molecular signalling,^11^ formation of cellular tight junctions,^12^ and cellular transport^13^ are other prime examples. These biomimetic setups are also used to understand active regulation of cellular processes by phase-separated liquid droplets. Examples include shaping vesicles by various types of active particles,^14,15^ and remodelling of biomimetic cells, synthetic vesicles,^5,16–20^ nucleoli, and germinal vesicles.^21^

Vesicle membranes are formed by the self-assembly of lipid molecules in aqueous solution, thereby separating an interior compartment from the surrounding bulk medium. These vesicles have a size that varies over a wide range from a few tens of nanometers, like synaptic and secretory vesicles, to hundreds of nanometers such as cellular organelles. Besides cellular vesicles, they also represent synthetic vesicles prepared from synthetic molecules such as block copolymers. Interior aqueous droplets have been found capable of modulating morphological transformations of vesicles.^4,6,7,22^ Conversely, lipid membranes can regulate the phase separation of biomolecular condensates.^23^ The experimental use of vesicles as model cells has mostly focused on giant vesicles on the micron scale to mimic the cell and cellular organelles.

Recent findings have highlighted the significant role of aqueous droplets in shaping biomembranes on the nanoscale, thereby regulating nano-sized cellular processes and structures. Formation of membrane nanotubes by phase separation of aqueous phases^7^ and autophagosome biogenesis in autophagy^24–26^ exemplify membrane remodeling by liquid nanodroplets. Membrane wetting by liquid droplets has been found to play a key role in forming the pre autophagosomal structure (PAS) as a precursor for autophagosome biogenesis in autophagy.^24–26^ The PAS is hypothesized to have formed *via* fusion of a few, reportedly three, Atg9 vesicles with a diameter within the range 30–60 nm resulting in a larger vesicle, 50 to 100 nm in diameter.^27^ How aqueous droplets regulate biological processes and shape cellular organelles, in particular on the nanoscale where experimental imaging becomes fragile, can be understood by simulating a nanovesicle with internal aqueous nanodroplets.

Based on theories of wetting and elastocapillary,^28,29^ vesicle remodelling by internal aqueous droplets depends on the interplay between four factors: the curvature elasticity of the vesicle; membrane tensions within membrane segments; interfacial tensions at liquid–liquid interfaces; and mostly-negative line tensions acting along the contact line of the vesicle and the droplets. Within the theoretical framework of curvature elasticity,^30–32^ membrane elastic energy depends on local or non-local spontaneous curvature. For simplified uniform membranes considered here, membrane shape is modulated by non-local spontaneous curvature or membrane asymmetry which is the difference in the lipid numbers between the two leaflets.^33–37^ It results from lipid exchange between the membrane leaflets, from non-uniform membrane growth by lipid synthesis, and from vesicle fusion. Interfacial surface and line tensions depend on the interfacial forces between the two aqueous phases *i.e.* liquid–liquid interactions represented in our model by the phase-separation propensity (referred to as separation propensity hereafter). The vesicle-droplet system, composed of a vesicle with two internal liquid phases of fixed concentration, is thus characterized by two properties: membrane asymmetry; and separation propensity between the liquid phases.

On the micron scale, the free energy contributions of membrane and interfacial tensions dominate those of the line tensions.^6,38,39^ Therefore, compared to membrane asymmetry and membrane and interfacial tensions, the line tensions are hypothesized to play a negligible role on the morphological transformations of giant vesicles wetted by large liquid droplets. By contrast, morphological behaviour of nanovesicles is expected to be significantly affected by the mostly-negative line tensions.^38^

Due to the small size of the nanovesicles and the dynamic nature of their shape transformations it is not easy to study their morphological behavior experimentally. Molecular dynamics simulation of coarse-grained models provides an alternative approach to explore both equilibrium and dynamic vesicle remodelling. We used DPD (Dissipative Particle Dynamics) simulations^40^ of a coarse-grained membrane model which has been successfully applied to study vesicle dynamics. Prime examples include the evolution of lipid vesicles and polymersomes under osmotic deflation,^41,42^ vesicle deformation by lipid flippase,^37,43,44^ vesicle fission by membrane phase separation,^45,46^ and vesicles in contact with aqueous droplets.^38^ Coarse-grained DPD simulations allow us to perform relatively long simulations of about 100 μs of rather large nanovesicles.

Our first goal is to explore vesicle compartmentation by non-equilibrium phase separation of two initially-mixed liquid phases inside an initial spherical vesicle. Then, we propose to investigate morphological behaviour of the resulting stable two-compartment and meta-stable multi-compartment vesicles under osmotic deflation to understand how membrane asymmetry and separation propensity determine the vesicle architecture. In our model the lipid membrane and the aqueous liquids were presented by coarse-grained beads and the phase-separation of liquids occurs due to steric repulsive interactions between them,^15^ where stronger repulsion corresponds to a larger separation propensity. See Methods for more details about our coarse-grained simulations.

We started by simulating non-equilibrium phase separation of two initially-mixed liquid phases forming both stable two-compartment and meta-stable multi-compartment nanovesicles. When we reduced the vesicle volume, meta-stable vesicles exhibited complex morphologies, such as branched tubules, with long lifetimes presumably due to the stabilizing role of the interfacial tensions. To understand complex polymorphism of mostly multi-compartment nanovesicles, we then focused on stable two-compartment vesicles as the building blocks of meta-stable multi-compartment nanovesicles. Upon volume reduction, mimicking the osmotic deflation and vesicle fusion in autophagy, these vesicles underwent different morphological transitions between stable tube, sheet and cup shapes, identified by membrane asymmetry and separation propensity. We demonstrated that lower membrane asymmetry, *i.e.* smaller non-local spontaneous curvature, and larger separation propensity promote formation of the sheet-like structures thereby transforming the vesicles from tubes to sheets and in turn toward cups.

## Methods

### DPD simulation

We used LAMMPS simulator^47^ to perform DPD simulations. The vesicles were built from lipid molecules composed of three hydrophilic head groups H and two hydrophobic chains of six tail groups C surrounded by an external liquid phase γ composed of beads of type D.^37^ All vesicles enclosed two internal liquid phases α and β corresponding to bead types A and B. All beads had a diameter of *d* = 0.8 nm which defined the length scale of our simulations.

The interaction force parameter *f*_ij_ between the liquid bead types A (α), B (β), and D (γ), and lipid head H and tail C groups. The interfacial force parameter *f*_AB_ varies in the range *f*_AB_ ∈ [35, 50]*k*_B_*T*/*d* to adjust *p*, see Table 2.

Interfacial force parameter *f*_AB_ between the two liquid phases with the corresponding separation propensity *p*.

Pairwise interactions between different pairs of DPD beads are distinguished by the interaction force parameter *f*_ij_ of the conservative DPD force.1
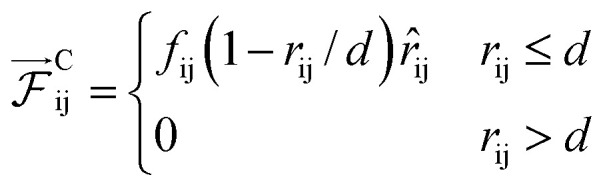


The unit vector *r̄*_ij_ points from bead i to bead j with a distance *r*_ij_. The remaining random and dissipative terms of the DPD pairwise force are identical for all bead pairs.

The two liquid phases were taken to equally wet the membrane for interfacial force parameters *f*_Ai_ = *f*_Bi_ (i = H and C) between the liquid beads A and B, and the lipid head H and hydrocarbon tail C groups. The external liquid phase γ, composed of bead type D, is also an aqueous phase with the same properties of the tow phases α and β.

The thermal energy *k*_B_*T*, obtained from Boltzmann constant *k*_B_ and absolute temperature *T*, defines the energy scale. All simulations were performed in an NVT ensemble in cubic simulation boxes with volume (80 *d*)^3^ under periodic boundary conditions. The DPD bead density 3/*d*^3^, together with an interaction force parameter *f*_AA_ = *f*_BB_ = 25 *k*_B_*T*/*d* leads to aqueous liquid phases that approximate water compressibility at room temperature. DPD equations were integrated with a time step 
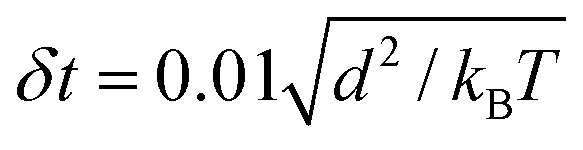
 where the time scale 
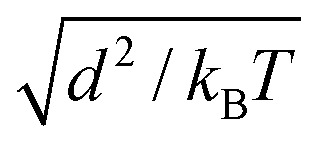
 of the system is about 1 ns for beads of unit mass. Except for interfacial force parameter *f*_AB_ which was varied to control the phase separation between the liquid phases α and β, the same interaction parameters, listed in Table 1, were used for all simulations throughout this article.

**Table 1:** Interaction force parameters *f*_ij_ [*k*_B_*T*/*d*]

	j = H	j = C	j = D	j = A	j = B
i = H	30	50	30	30	30
i = C	50	10	75	75	75
i = D	30	75	25	25	25
i = A	30	75	25	25	*f*_AB_
i = B	30	75	25	*f*_AB_	25

### Interfacial surface tension and phase separation propensity

To find the interfacial surface tensions, a slab of liquid phase β was placed in the middle of a periodic simulation box surrounded with liquid phase α at both sides, see Fig. S1A.† The interfacial tension depends on the repulsive interactions between the two liquid phases *i.e.* their tendency to phase separate. Liquid phase separation between the liquid phases α and β is defined by the rescaled separation propensity *p* = *f*_AB_/*f*_AA_ as the ratio of the interfacial force parameter *f*_AB_ and the internal force parameter *f*_AA_ = *f*_BB_ = 25 *k*_B_*T* within each liquid phase. The separation propensity *p* = 1.48, corresponding to the interfacial force parameter *f*_AB_ = 37 *k*_B_*T*, was obtained as the particular interfacial force for which the density of each liquid phase was above 95% of the total DPD bead density 3/d^3^, see Fig. S1C.† Below this separation propensity, the two liquids completely mixed including the smallest propensity *p* = 1.4, which we used for two miscible liquid phases. In this article, we used five different interaction force parameters *f*_AB_ with their corresponding separations propensities as listed in Table 2.

**Table 2:** Separation propensities *p* for different interfacial force parameters *f*_AB_

*f*_AB_ [*k*_B_*T*/*d*]	*p*
35	1.4
37	1.48
40	1.6
45	1.8
50	2

The interfacial tension *Σ*_αβ_ was computed by integrating the Cartesian stress profile2
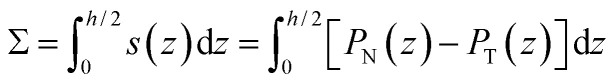


perpendicular to the interfacial surface of the two liquids.^38,48,49^ Here, *P*_N_(*z*) and *P*_T_(*z*) are the normal and tangential pressure components and *h* is the height of the simulation box, see Fig. S1A.† The smallest separation propensity *p* = 1.4 corresponds to two miscible liquid phases that do not phase-separate based on the criterion we used here. Larger separation propensities 1.48 < *p* ≤ 2 corresponding to larger interfacial tensions *Σ*_αβ_, lead to stronger more active liquid phase separation, see Fig. S1B† and Table 3. We thus found that the interfacial tensions depend on the separation propensity *p* an are independent of *Δ*.

**Table 3:** Interfacial tension *Σ*_αβ_ and line tension *λ*

*p*	*Σ*_αβ_ [*k*_B_*T*/*d*^2^]	*λ* [*k*_B_*T*/*d*]
1.48	0.45 ± 0.09	− 0.21 ± 0.43
1.6	0.87 ± 0.13	− 0.77 ± 0.39
1.8	1.4 ± 0.13	− 1.81 ± 0.38
2	1.88 ± 0.13	− 2.63 ± 0.37

### Assembling initial spherical vesicles

**Fig. 1:**
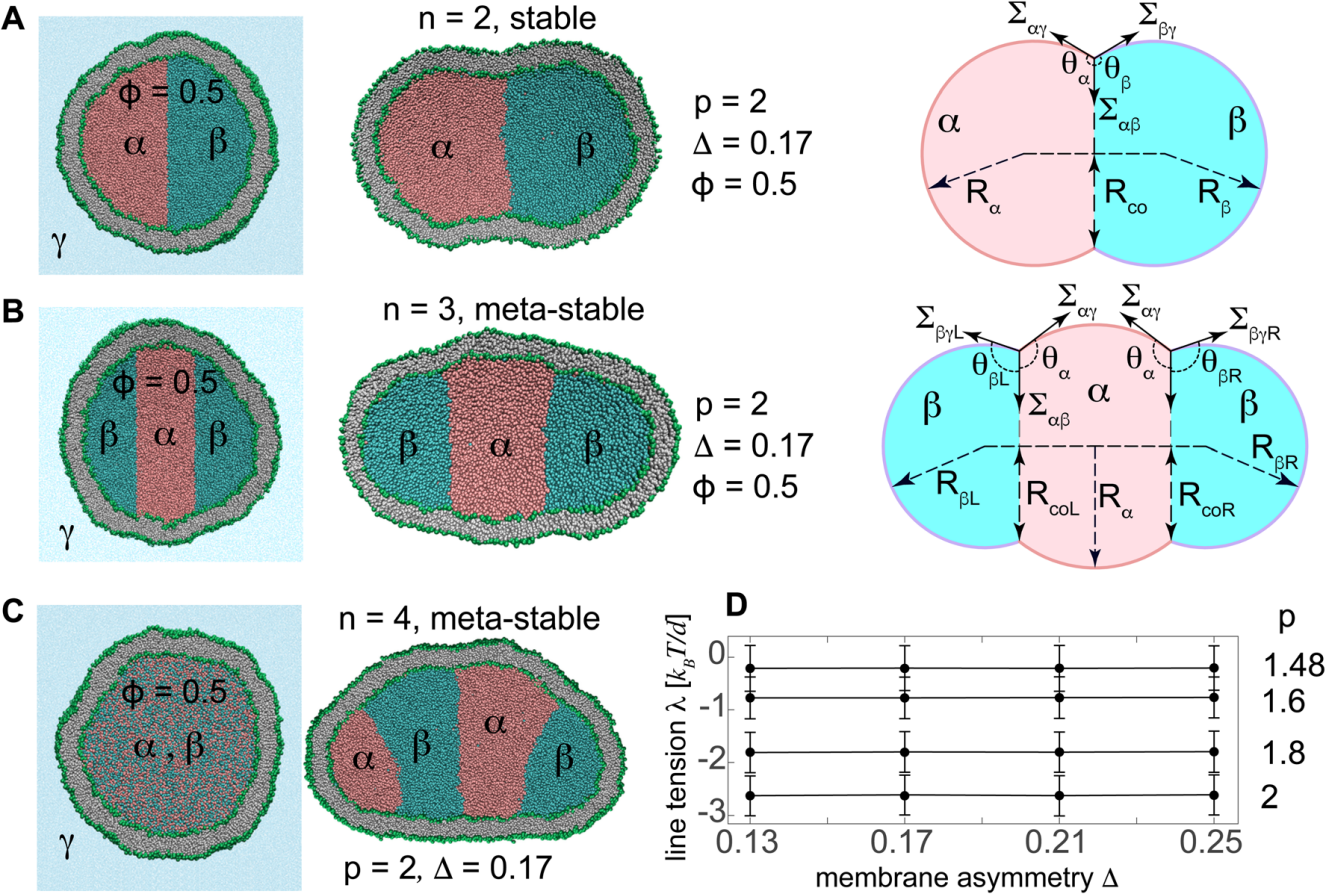
(A and B) Initial spherical and equilibrated small vesicles with fixed *N*_lip_ = 12 000 and *Δ* = 0.17 enclosing an initial volume *v* = 1 of (A) two and (B) three separated liquid droplets composed of liquid phases α (pink) and β (cyan) with *ϕ* = 0.5 and *p* = 2 surrounded by liquid phase γ (blue). The right panels show the schematic diagram of the stable/meta-stable vesicles with two/three compartments for *ϕ* = 0.5 composed of spherical caps with radii *R*_α_ and *R*_β_, and having interfacial and membrane tensions, *Σ*_αβ_, *Σ*_αγ_, and *Σ*_βγ_. Subscripts *R* and *L* denote right and left droplets for the three-compartment vesicle in (B). (C) Initial and equilibrated vesicles with initially randomly mixed liquid phases, separated into *n* = 4 droplets. (D) Mostly-negative line tensions λ of the vesicles are almost independent of *Δ* and depend on *p*. *λ* acts along the contact circle of radius *R*_co_.

We first assembled spherical vesicles with a fixed total number *N*_lip_ = 12 000 of lipids by placing *N*_i_ = 5220 and *N*_o_ = *N*_lip_ − *N*_i_ = 6780 lipids with their heads on two concentric spheres of radii *R*_i_ = 19*d* and *R*_o_ = 25*d*, corresponding to inner and outer leaflets respectively. The length scale *d* ≃ 0.8 nm denotes a single bead diameter. These vesicles, nearly 32 nm in diameter, initially enclose *N*^i^_liq_ = *N*^i^_A_ + *N*^i^_B_ = 126 000 liquid beads of types A and B corresponding to liquid phases α and β with relative concentration *ϕ* = *N*^i^_A_/*N*^i^_liq_ which defined the initial vesicle volume. The two liquid phases are either initially separated, see Fig. 1A and B, or are randomly mixed as seen in the left panel of Fig. 1C. The surrounding liquid phase γ is an inert spectator phase, see Fig. 1A. Upon volume reduction, a vesicle with reduced volume *v* = *N*_liq_/*N*^i^_liq_ ≤ 1 contains less number *N*_liq_ < *N*^i^_liq_ of liquid beads for a constant relative concentration *ϕ* = *N*_A_/*N*_liq_ = *N*^i^_A_/*N*^i^_liq_.

For the given total number *N*_lip_ = 12 000 of lipids, we redistributed the lipids between the two leaflets to produce five vesicles with different membrane asymmetries *Δ* = (*N*_o_ − *N*_i_)/*N*_lip_ = 0.13, 0.17, 0.21, 0.25, and 0.27, representing the relative difference in lipid numbers of the two vesicle leaflets. These vesicles were then equilibrated with the two internal nanodroplets of relative concentration *ϕ* = 0.5 and different separation propensities. Vesicle parameters of the resulting vesicles are listed in Tables S1 to S3.†

To determine the reference state of membrane asymmetry, we simulated the vesicle with a single internal liquid phase α, see Fig. S2A.† For the vesicle with *N*_lip_ = 12 000, a unique combination of *N*_i_ = 5023 and *N*_o_ = 6977 leads to a reference vesicle, with *Δ*_r_ = 0.16, for which both leaflets experience equal surface tensions,^37^ shown to vanish upon a small reduction in the vesicle volume as shown in Fig. S2B.† The reference vesicle is characterized by its negligible non-local spontaneous curvature. Lipid chains were taken long enough to suppress lipid exchange between the leaflets, resulting in a constant membrane asymmetry *Δ* for each individual vesicle. Fig. S2C† shows the leaflet tensions of this vesicle as well as other initial vesicles with different membrane asymmetries whose leaflets have different tensions and are thus expected to show different morphological behaviour under volume reduction. Vesicles with membrane asymmetries different from that of the reference vesicle *Δ* ≠ *Δ*_r_ = 0.16 have their outer and inner leaflets stretched or compressed depending on larger or smaller asymmetries relative to the reference state *Δ*_r_ = 0.16.

### Membrane tensions

The two liquid phases were taken to equally wet the membrane with identical molecular interactions between liquid beads A and B and both lipid head H and hydrocarbon tail C groups resulting in identical affinities of both liquid phases for the vesicle bilayer. The vesicle bilayer thus experiences a uniform membrane tension *Σ* = *Σ*_αγ_ = *Σ*_βγ_ across its surface.

Line and interfacial tensions as a function of *p* for a stable vesicle with two compartments according to the computational method presented in Fig. 1. Both line and interfacial tensions depend on *p*.

Membrane tension within different membrane segments was computed by finding the first moment of the stress profile across the membrane interface. For initial spherical vesicles of unit volume *v* = 1 with a single internal liquid phase and different membrane asymmetries *Δ*, the stress or pressure profile has spherical symmetry. The normal and tangential pressure components *P*_N_(*r*) and *P*_T_(*r*) are thus functions of the radial coordinate *r*. For these vesicles, the bilayer tension *Σ* was obtained by integrating the stress profile *s*(*r*) = *P*_N_(*r*) − *P*_T_(*r*) across the membrane.3
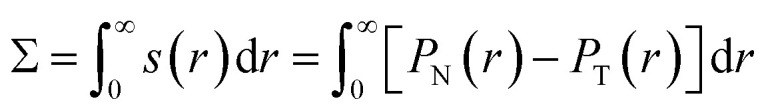


The two leaflet tensions were also obtained by splitting the same integral at the bilayer midplane *r* = *R*_m_ as4
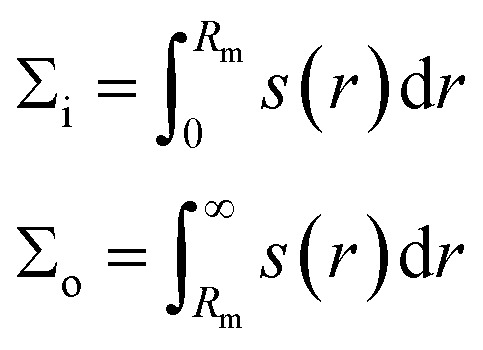
for inner and outer leaflets, respectively. Membrane tensions in nonspherical vesicles were calculated by integrating the Cartesian pressure components *P*_N_ = *P*_zz_ and *P*_T_ = 0.5(*P*_xx_ + *P*_yy_) across each membrane segment separating a liquid droplet of each phase α, β from external bulk liquid phase γ. The pressure components were computed inside a rectangular box along the *z* axis normal to the bilayer surface, see Fig. S3B.† Membrane tensions, calculated in this way, mainly depend on the molecular interactions and are thus identical across all membrane interfaces αγ and βγ in our multi-compartment vesicles.

### The free energy of the vesicles and the line tension

To compute the line tensions, we started from two initially-separated liquid nanodroplets inside the vesicle with *Δ* = 0.17, known to have a negligible non-local spontaneous curvature.

The free energy.

Two and three initially-separated liquid droplets inside an initially-spherical reference vesicle, Fig. 1A and B left panels, rapidly transformed into stable and meta-stable vesicles with *n* = 2 and 3 droplets respectively, Fig. 1A and B middle panels. The free energies *F*_2_ and *F*_3_ of these vesicles are5
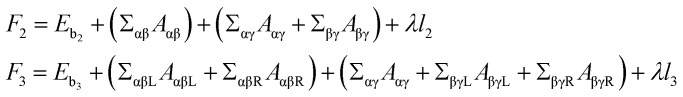
composed of vesicle bending energies *E*_bi_ (*i* = 2, 3), interfacial tension energies (first parentheses), and membrane-tension energies (second parentheses). Here, *λ* is the line tension, *Σ*_αβ_ is the interfacial tension, and *Σ*_αγ_ and *Σ*_βγ_ are membrane tensions. As depicted in the right panels of Fig. 1A and B, the architecture of each nanovesicle with two or three internal nanodroplets, found by fitting spherical caps to membrane segments, determines the interfacial and membrane areas *A*_ij_ (i, j = α, β, γ), the length *l*_*i*_ (*i* = 2, 3) of the contact line along which the line tension acts, and the bending energies *E*_bi_ of membrane segments as given in ESI eqn (S2) and (S3).†

The line tension.

The two internal liquid phases α and β are separated from the external aqueous phase γ through membrane interface. The intersection of the interfacial surface αβ and the membrane defines a triple phase line along which the line tension acts. Initially-spherical vesicles were shortly equilibrated with a single internal liquid phase which was subsequently divided into two separate phases with relative concentration *ϕ* = 0.5, see the left panel in Fig. 1A. Two initially-separated liquids with different separation propensities *p*, and corresponding interfacial tension *Σ*_αβ_, rapidly equilibrated into a stable two-compartment vesicle, with minimum free energy as seen in the right panel of Fig. 1A. Knowing the interfacial and membrane tensions for the given separation propensity *p*, we computed the line tension as described in more details in the ESI S1.† We then varied the separation propensity *p* inside different vesicles with various membrane asymmetries and found the corresponding line tensions. Like interfacial tensions, line tensions were found to depend on the separation propensity *p* and not on *Δ*, see Fig. 1D and Table 3.

## Results and discussion

We first assembled initial vesicles with different membrane asymmetries enclosing two liquid phases with various separation propensities that were either initially separated or mixed. In this way, each stable vesicle was characterized by two independent parameters: the separation propensity *p* which exclusively determines the pair of interrelated line tension *λ* and interfacial tension *Σ*_αβ_; and the membrane asymmetry *Δ*. These vesicles are subsequently used to simulate vesicle compartmentation and morphological transition of compartmetalized vesicles under osmotic deflation.

### Vesicle compartmentation by liquid phase separation

To explore the polymorphism of both stable and meta-stable vesicles, we started with non-equilibrium phase separation of two initially-mixed liquids by steric interactions.^15^ The initial mixture was assembled by random distribution of liquid beads α and β with relative concentration *ϕ* inside an initially-spherical vesicle with *v* = 1, see the left panel of Fig. 1C.

For each set of *ϕ* and *p*, thirty simulations were performed from different initial random arrangements of liquid beads A and B (see the left panel in Fig. 1C) for a short time, *t* = 5 μs, and the statistical distribution of number of compartments were obtained.

**Fig. 2:**
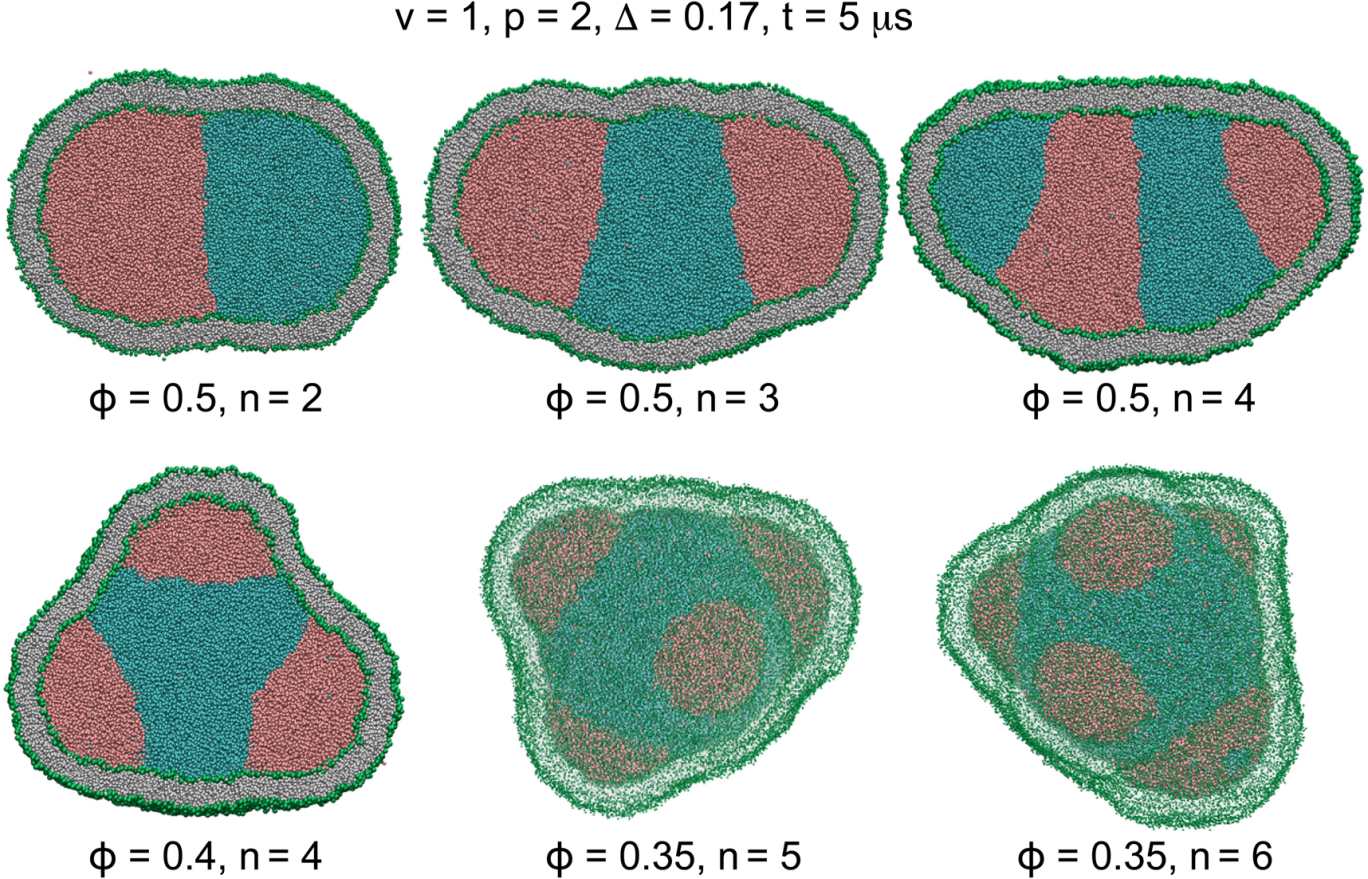
Cross sections (*n* = 2–4) and 3D views (*n* = 5 and 6) of actively-compartmentalized stable two-compartment (*n* = 2) and meta-stable multi-compartment vesicles (*n* > 2) with *Δ* = 0.17, having a negligible non-local spontaneous curvature, obtained from phase separation of initial random mixtures of the two liquids with *p* = 2 and different values of *ϕ* inside initially-equilibrated vesicles with *v* = 1 as seen in Fig. 1C. Number of compartments n decreases with *ϕ* and increases with *p* as shown in Table 4. The snapshots show the vesicles after a rather short simulation time of *t* = 5 μs when the two liquids have separated.

Despite the entropic forces that tend to retain the mixture, the two liquids rapidly phase-separated due to steric forces between them. The non-equilibrium phase separation of the internal liquids thus transformed the vesicle shape by exerting active forces from liquid droplets on the vesicle membrane. Steric interactions between the two liquid phases were controlled by separation propensity *p* which determines the compartmentation activity level. These active forces compartmentalized the vesicle by forming various multi-droplet structures inside the nanovesicles with *n* compartments some of which are displayed in Fig. 2 and Video SV4.†

For each pair of relative concentration *ϕ* ∈ {0.4, 0.5} and separation propensity 1.6 ≤ *p* ≤ 2, we performed thirty simulations starting from different initial random assembly of liquid beads. For two liquids equally wetting the membrane, the liquid phase with smaller concentration rapidly nucleated into multiple nanodroplets, in about 5 μs, on the periphery of a single droplet of the liquid phase with larger concentration. Both stable two-compartment, *n* = 2, and meta-stable multi-compartment vesicles, *n* > 2, resulted from vesicle compartmentation, as listed in Table 4 and shown in Fig. 2. The number of nanodroplets, and thus the vesicle compartments *n*, increased with the separation propensity *p* and decreased with the relative concentration *ϕ*. More active compartmentation with larger separation propensity *p* led to nanovesicles with more compartments giving rise to an enriched polymorphism of the resulting membrane structures.

**Table 4:** Number of compartments *n* inside vesicles

*ϕ*	*p*	*n* = 2	*n* = 3	*n* ≥ 4
0.5	1.6	77%	23%	—
0.5	2	66%	30%	4%
0.4	1.6	16%	68%	16%
0.4	2	10%	47%	43%

For *n* > 2, the small nanodroplets are expected to coalesce to form two large droplets of the two liquid phases to reduce the free energy contributions of the interfacial tensions, membrane tensions, and membrane bending, as reflected in eqn (6). Interestingly, however, as seen in Video SV5,† we did not observe droplet coalescence in our extended simulation, for about 100 μs, of a meta-stable nanovesicle with three separated nanodroplets, with membrane asymmetry *Δ* = 0.17 and line and interfacial tensions *λ* = −2.6 *k*_B_*T*/*d*, *Σ*_αβ_ = 1.88 *k*_B_*T*/*d*^2^.

**Fig. 3:**
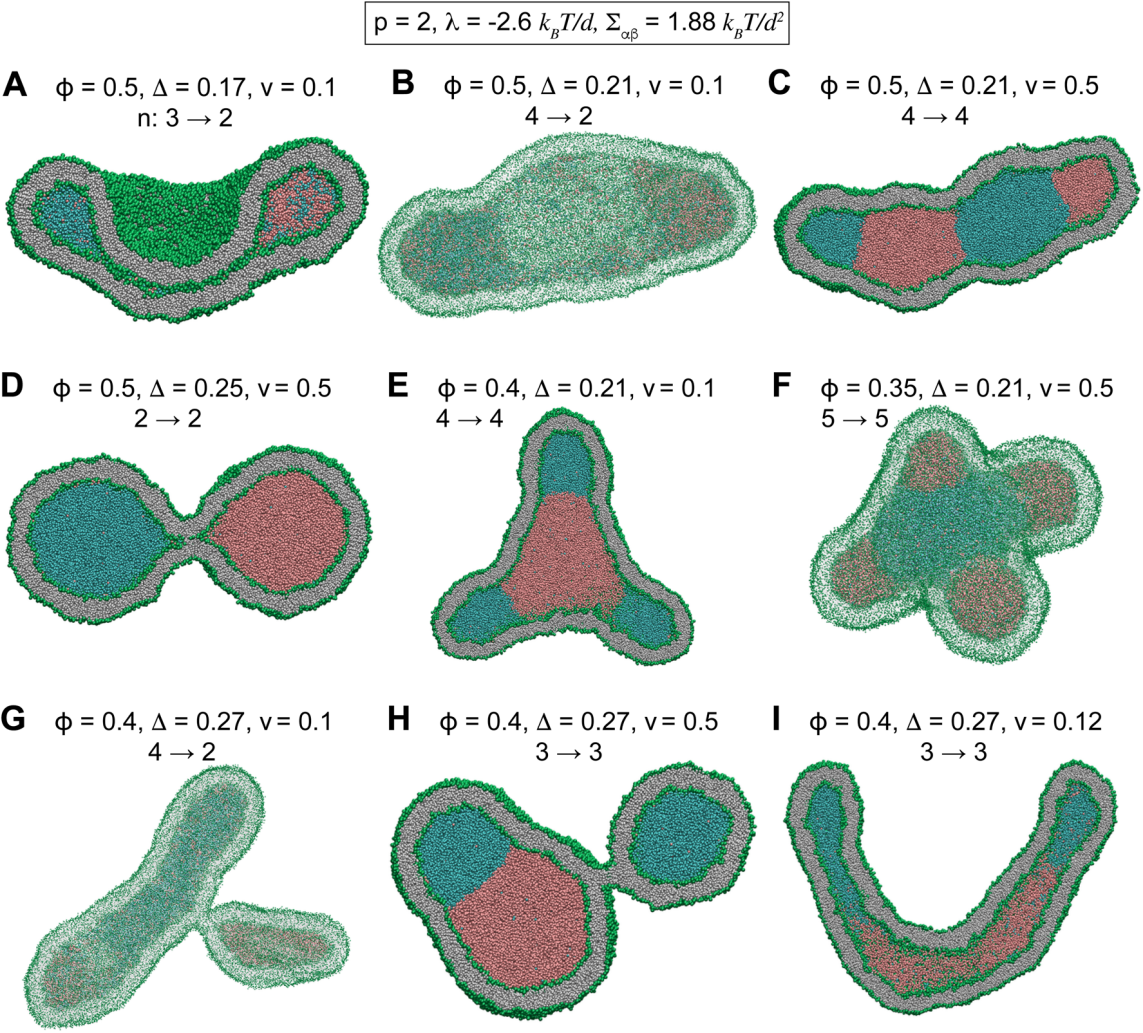
Morphological transformations of stable and meta-stable compartmentalized vesicles with different values of *ϕ*, *v*, and *Δ* for a fixed *p* = 2, corresponding to *λ* = −2.6 *k*_B_*T*/*d* and *Σ*_αβ_ = 1.88 *k*_B_*T*/*d*^2^. (A) A cup-shaped vesicle at small *Δ* = 0.17. (B) A meta-stable sheet structure. (C) A meta-stable long-lived tubular vesicle. (D) A stable dumbbell with circular neck at high *Δ* = 0.25. (E) A meta-stable branched tubule. (F) A meta-stable five-compartment vesicle. (G) A meta-stable branched tubule. (H) A meta-stable three-compartment vesicle. (I) A meta-stable long tubule at high *Δ* = 0.27. The number *n* of the initial and final compartments of the vesicles are shown by arrows above each vesicle. Morphological transitions of multi-compartment vesicles.

Starting from a nanovesicle with interior phase-separated multi-droplets, we slowly reduced the vesicle volume from *v* = 1 to *v* = 0.1 in about 90 μs. In all the following simulations, the vesicle volume is reduced by a fixed rate of 0.01 per micro second which is sufficiently small to ensure equilibrium intermediate vesicles.^37^Fig. 3 and S4† show some of intermediate vesicle structures with various values of *ϕ* and *v*. Morphological evolution of some of these vesicles through intermediate morphologies is shown in Videos SV6 to SV8.† Vesicles were observed to undergo shape transitions consisting a rich variety of both stable two-compartment and meta-stable multi-compartment conformations such as branched tubules (Fig. 3E, G and S4F†), dumbbells (Fig. 3D), tubes (Fig. 3I), sheets (Fig. 3B), cups (Fig. 3A), and pearled vesicles (Fig. 3C and H) with up to *n* = 5 (Fig. 3F and S4C†) and *n* = 6 (Fig. S4E†) compartments. Whereas some of the internal droplets of multi-compartment vesicles coalesced to form larger droplets upon vesicle deflation, many others persisted over long 90 μs simulations.

### Stable two-compartment vesicles

**Fig. 4:**
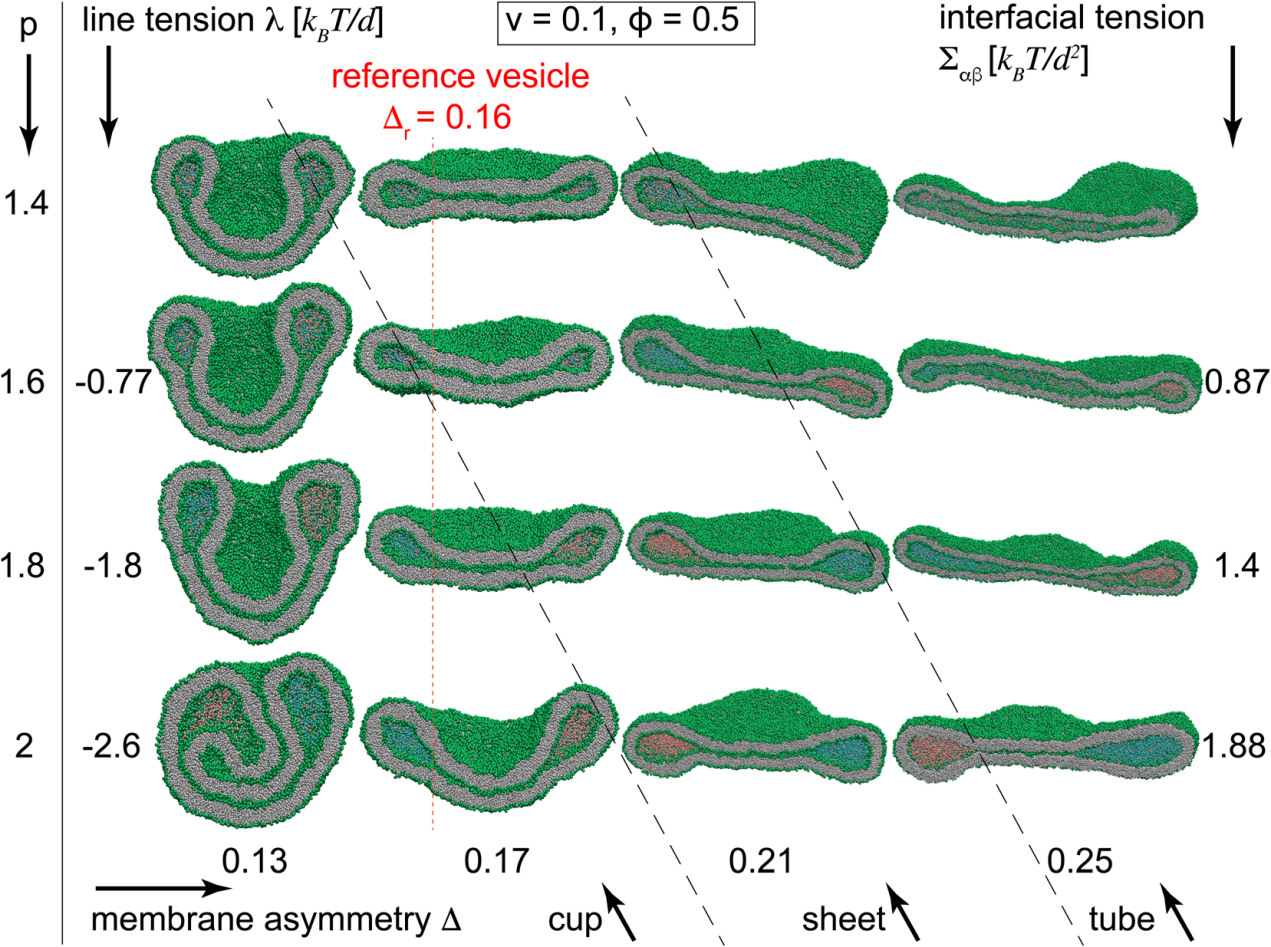
Morphological diagram of the stable vesicles with *n* = 2 liquid compartments at low volume *v* = 0.1 for different separation propensities 1.4 ≤ *p* ≤ 2, corresponding to −2.6 *k*_B_*T*/*d* ≤ *λ* ≤ 0 (rows) and 0.13 ≤ *Δ* ≤ 0.25 (columns). The black dashed lines show approximate transitions between cup, sheet and tubular vesicle morphologies. More negative line tensions and smaller membrane asymmetries transform vesicles from tubules into sheets and eventually towards cups. At constant *Δ*, more negative line tensions result in transition from sheet to cup as seen in the two left most columns with *Δ* = 0.13 and 0.17. The second column with *Δ* = 0.17 shows the closest vesicles to the reference states of *Δ*_r_ = 0.16 for which both leaflets have equal tensions. The first row represents vesicles with two miscible liquid phases with no contact line corresponding to *p* = 1.4.

To better understand the novel membrane nanostructures of meta-stable multi-compartment nanovesicles, we concentrated on two-compartment nanovesicles as the building blocks of the more general multi-compartment vesicles. Starting from equilibrated vesicles with various membrane asymmetries 0.13 ≤ *Δ* ≤ 0.25 and two initially-separated liquid droplets, we slowly decreased the vesicle volume to *v* = 0.1, thereby constructing the morphological diagram of stable two-compartment vesicles. Simulations were performed for vesicles with internal liquid phases with *ϕ* = 0.5 and different separation propensities 1.48 ≤ *p* ≤ 2, corresponding to −3 *k*_B_*T*/*d* < *λ* < 0 and *Σ*_αβ_ ∈ (0.4, 2)*k*_B_*T*/*d*^2^. We also simulated particular vesicles with miscible internal liquids with *p* = 1.4, for which the line and interfacial tensions are not defined. We observe morphological transitions between tubules, sheets, and cup vesicles for different values of *Δ* and *p*, see Fig. 4 and Videos SV1 to SV3.†

Inspecting columns of Fig. 4, we observe transitions from mostly tubular structures for large membrane asymmetries *Δ* = 0.25, into sheets for intermediate asymmetries *Δ* = 0.17 and 0.21, and toward cups for *Δ* = 0.13 at the leftmost column. For any individual column with a fixed *Δ*, more negative line tensions and larger interfacial tensions, corresponding to larger *p*, transform the vesicle from mostly tubular to mostly sheet-like structures as seen for *Δ* = 0.21 and 0.25. For smaller membrane asymmetries *Δ* = 0.13 and 0.17, a similar transition from sheet-like to cup-shaped vesicles takes place upon decreasing line tensions to more negative values. Similar behaviour is observed for the special case of *p* = 1.4, corresponding to two fully miscible liquids without energetic contributions from line and interfacial tensions, see first row in Fig. 4. As seen in the bottom left snapshot of Fig. 4, larger separation propensity *p* = 2 corresponding to a larger *Σ*_αβ_ = 1.88 *k*_B_*T*/*d*^2^ and a more negative *λ* = −2.6 *k*_B_*T*/*d* at sufficiently low *Δ* = 0.13 are even capable of forming a double-cup morphology.

The key step in forming membrane stacks and cup membranes, critical for many biological processes and ubiquitous in cellular membranes, is the formation of intermediate sheet morphology. For stable vesicles with constant vesicle volume, the positive interfacial tension tends to reduce the area of the interfacial surface while the negative line tension acts to increase its perimeter *i.e.* the length of the contact line. The two effects cooperate to form lipped-like structure, a characteristic feature of sheet membranes, that helps transform tubular structures toward sheet membranes which in turn bend toward the cup shape for more negative line tensions or lower membrane asymmetries.

**Fig. 5:**
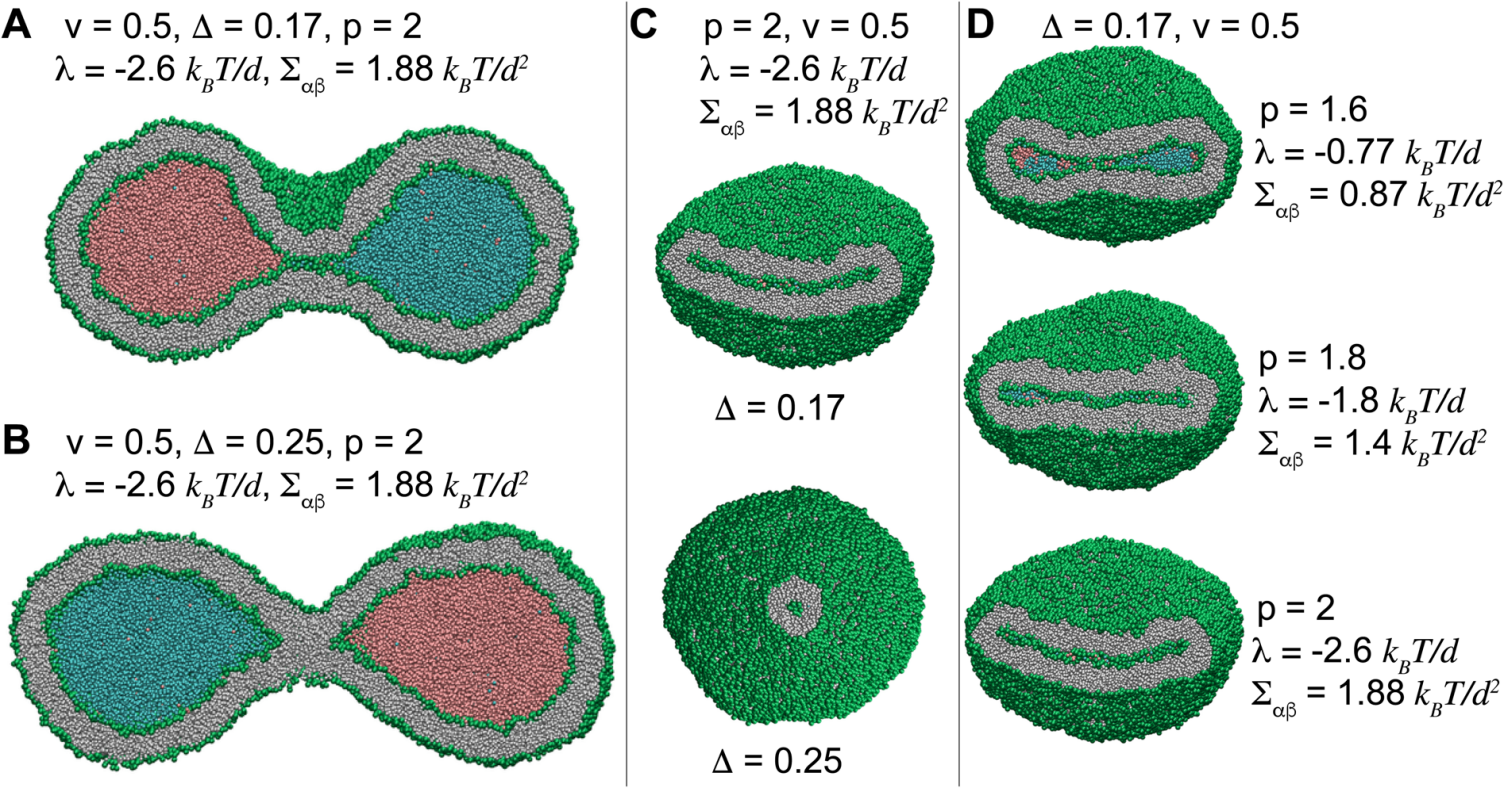
A stable dumbbell vesicle of volume *v* = 0.5 with two liquid droplets with *ϕ* = 0.5 and *p* = 2, corresponding to *λ* = −2.6 *k*_B_*T*/*d* with (A) *Δ* = 0.17 and (B) *Δ* = 0.25, mimicking cell division and suggesting a mechanism for controlled division of synthetic vesicles. (C) Vesicles with volume *v* = 0.5 and *λ* = −2.6 *k*_B_*T*/*d* cut at the interface between the liquids. The lipped-like section of a sheet for *Δ* = 0.17 transforms to the circular cross section of a local tubule for *Δ* = 0.25. (D) The cross section of a dumbbell vesicle at the interfacial surface with constant *Δ* = 0.17 and volume *v* = 0.5 transforms from a sheet-like structure for a small *λ* = −0.77 *k*_B_*T*/*d* to a wider sheet for *λ* = −0.77 *k*_B_*T*/*d* that bends for more negative *λ* = −2.6 *k*_B_*T*/*d*. The three cross sections show how a biconcave structure in the top snapshot transforms into a convex-concave shape for more negative line tensions, with the latter one being a prerequisite for the formation of the cup structure.

Fig. 5 displays how a stable dumbbell vesicle with two compartments (Fig. 5A and B) adopts sheet-like structure at the interfacial surface for a more negative *λ* and a larger *Σ*_αβ_ (Fig. 5D) at fixed *Δ* = 0.17. A similar sheet-like cross section is observed in Fig. 5C for smaller *Δ* = 0.17 at constant *λ* = −2.6 *k*_B_*T*/*d* and *Σ*_αβ_ = 1.88 *k*_B_*T*/*d*^2^, compared to the circular cross section for *Δ* = 0.25.

### The instability of multi-compartment vesicles

Multi-compartment giant vesicles are known to be meta-stable as energetic penalties of membrane tensions make vesicles with two coalesced droplets more favorable than vesicles with multi-droplets. We need, however, to examine the stability of nanovesicles in presence of negative line tensions we have already found to contribute to the shapes of the nanovesicle. To explore the stability of multi-compartment vesicles, we focused on two vesicles with *Δ* = 0.17, close to the reference vesicle *Δ*_r_ = 0.16, and thus negligible non-local spontaneous curvature. The liquid phases were taken to strongly phase separate with *p* = 2 corresponding to *Σ*_αβ_ = 1.88 *k*_B_*T*/*d*^2^ and *λ* = −2.6 *k*_B_*T*/*d*. For the two vesicles with *n* = 2 and 3 compartments, see Fig. 1A,B middle panels, the free energies are given by6
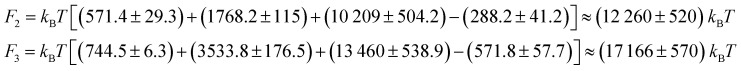
where the free energy *F*_2_ = 12 260 *k*_B_*T* of the stable two-compartment vesicle with *n* = 2 is much less than that of the meta-stable three-compartment vesicle *F*_3_ = 17 166 *k*_B_*T*. Membrane and interfacial tensions favor the two-compartment vesicle, increasing from *F*_2_ to *F*_3_ by (3533.8 − 1768.2) *k*_B_*T* = 1766 *k*_B_*T* and (13 460 − 10 209) *k*_B_*T* = 3251 *k*_B_*T* respectively, while negative line tension tends to stabilize the meta-stable three-compartment vesicle, decreasing by [ −288.2 − (−571.8)] *k*_B_*T* = 283.6 *k*_B_*T* from *F*_2_ to *F*_3_. As expected, the vesicle bending energy also tends to stabilize the two-compartment vesicle with a slight energy deference *E*_b_3__ − *E*_b_2__ = 173.1 *k*_B_*T*, which is due to the reduced area of the highly-curved spherical membrane segments formed for *n* = 3 droplets.

More strongly separating liquids with a larger *p* have larger interfacial tensions and more negative line tensions. While larger interfacial tensions stabilize two-compartment vesicles, more negative line tensions favor meta-stable three-compartment vesicles. The interfacial surface area 4π*R*_co_^2^, however, grows faster than the line 2π*R*_co_, with *R*_co_ being the radius of almost circular interfacial surface, see right panel in Fig. 1A. Negative line tensions are thus more likely to stabilize nanovesicles for which the contribution of these tensions becomes more comparable to those of the interfacial tensions as opposed to larger vesicles where surface tensions are dominant.

## Conclusion

Using extensive molecular dynamics simulations, we explored compartmentation of nanovesicles by liquid phase-separation and vesicle remodelling by the resulting aqueous droplets under osmotic deflation. We showed that the phase-separation propensity of the two liquid phases determines the line and interfacial tensions as well as the activity level of the vesicle compartmentation. The vesicles are thus parametrized by two independent parameters: membrane asymmetry — or its non-local spontaneous curvature — and the separation propensity. We identified morphological transitions of stable two-compartment vesicles between tubules, sheets, and cups, induced by liquid droplets and regulated by membrane asymmetry and separation propensity.

Our results elucidate how tubules, sheets, and cups, as the building blocks of cellular membranes can be induced and maintained by phase-separated aqueous nanodroplets. These structures, exclusive to nanovesicles, have important implications for shaping cellular membrane nanostructures. We find that typical negative line tensions, resulting from larger separation propensities, play a key role in sculpting nanovesicles. At fixed vesicle volumes, these negative tensions, together with positive interfacial tensions, lead to the formation of lipped-like cross sections, thereby transforming tubular shapes into sheet-like structures on stable vesicles with two compartments.^38^ Larger interfacial tensions and more negative line tensions can bend the sheet-like biconcave nanovesicle into concave-convex sheet, thereby forming the cup-shaped vesicles that even turn to double cups for larger separation propensities. For a fixed line tension, similar behaviour is observed by decreasing membrane asymmetries, or non-local spontaneous curvatures, where vesicles transform from tubules to sheets and eventually cup morphologies for smaller membrane asymmetries.

Quantifying the free energies of two stable and meta-stable nanovesicles with two and three compartments we demonstrated the instability of long-lived multi-compartment nanovesicles. We thus indicate the important role of internal nanodroplets in conferring meta-stable nanovesicles their prolonged lifetime. Phase-separated nanodroplets can thus contribute to creating and maintaining meta-stable membrane nanostructures such as branched tubules which serve as building blocks of many cellular organelles such as endoplasmic reticulum and mitochondria.

We also show how phase separation of liquids, with smaller relative concentrations and larger separation propensities, generates more compartments which subsequently lead to a more diverse polymorphism of nanovesicles upon osmotic deflation. For meta-stable multi-compartment vesicles with more than two compartments subject to volume reduction, more negative line tensions and higher asymmetries promote formation of meta-stable complex morphologies such as tubular branches. Some of these long-lived structures are also experimentally observed, for instance in nucleoli.^21^

Our results on forming cup-shaped vesicles have immediate implication for the recently-found impact of liquid phase separation in autophagosome biogenesis.^24–26^ Our findings also reveal that stable dumbbell vesicles with two aqueous compartments can be formed by adjusting appropriate combinations of separation propensities and membrane asymmetries. This suggests a novel mechanism that can be used to divide a vesicle into two smaller vesicles by internal liquid droplets, mimicking cell division, and may have particular applications in synthetic biology.^5^

Several structures, observed here for small vesicles, were previously reported for larger vesicles with liquid droplets including shape transformation of nucleoli,^3,21^Fig. 3C and H and S4A and D,† and division and budding of model cells and giant vesicles,^5,6,16,50^Fig. 5A, B and 3D.

## Conflicts of interest

There are no conflicts to declare.

## Author contributions

Fatemeh Kazemi Sabet: data curation, formal analysis, investigation, software, validation, visualization, writing – review &editing; Arash Bahrami: supervision, project administration, writing – review & editing; Amir H. Bahrami: conceptualization, formal analysis, investigation, methodology, project administration, resources, supervision, validation, visualization, writing – original draft preparation, writing – review & editing.

## Supplementary Material

RA-012-D2RA05855C-s001

RA-012-D2RA05855C-s002

RA-012-D2RA05855C-s003

RA-012-D2RA05855C-s004

RA-012-D2RA05855C-s005

RA-012-D2RA05855C-s006

RA-012-D2RA05855C-s007

RA-012-D2RA05855C-s008

RA-012-D2RA05855C-s009
