# Supplementary material for: Compartmentalizing and sculpting nanovesicles by phase-separated aqueous nanodroplets
Source: RSC Adv. 2022 Nov 8;12(49):32035–45. doi: 10.1039/d2ra05855c (PMC9642337; doi:10.1039/d2ra05855c)
Supplement: RA-012-D2RA05855C-s001 [file RA-012-D2RA05855C-s001.pdf]

Electronic Supplementary Material (ESI) for RSC Advances.
This journal is © The Royal Society of Chemistry 2022

Supporting Information: Compartmentalizing and Sculpting Nanovesicles by Phase-separated Aqueous Nanodroplets

Fatemeh Kazemi Sabet [†], Arash Bahrami [†] and Amir H. Bahrami [*,‡,¶]

†*School of Mechanical Engineering, College of Engineering, University of Tehran, North Kargar St., 14399-57131 Tehran, Iran*

‡*Bilkent University UNAM – National Nanotechnology Research Center, Ankara 06800, Turkey*

¶*Living Matter Physics, Max Planck Institute for Dynamics and Self-Organization (MPIDS), 37077 Göttingen, Germany*

E-mail: bahrami@unam.bilkent.edu.tr

Supplementary Text

S1. The line tension

A two-compartment vesicle with initially-separated liquid phases of relative concentration $\phi = 0.5$ was used to compute the line tensions. The vesicle rapidly equilibrated into a stable vesicle with minimum free energy. The interfacial tension $\Sigma_{\alpha\beta}$ between the two droplets for various vesicles was obtained previously as a function of p as described in the main text and presented in Figure S1 and Table S1. Membrane tensions, $\Sigma_{\alpha\gamma}$ and $\Sigma_{\beta\gamma}$, were also found by integrating the stress profile across the two membrane segments as shown in the top panel in Figure S3B and described in the main text. The vesicle volume V remains constant during equilibration and is thus identical for the initial spherical vesicle and the final equilibrated vesicle, see left and middle panel in Figure 1A. For a given set of interfacial surface and membrane tensions $\Sigma_{\alpha\beta}, \Sigma_{\alpha\gamma}, \Sigma_{\beta\gamma}$, the area of the initial spherical vesicle slightly increased to form the equilibrated vesicle with area A and an excess area $A_r = A^{3/2}/(6V\sqrt{\pi}) > 1$ for which the equilibrated two-compartment vesicle has minimum free energy $F_2 = F_2(A_r)$, see Figures 1A and S2A. The excess area A_r uniquely defines the shape of an equilibrated vesicle, for fixed droplet volumes $V_1 = V_2 = 0.5V$ corresponding to $\phi = 0.5$, by solving a nonlinear system of equations:

$$
\begin{aligned}
A &= 2\pi R_\alpha^2(1-\cos\theta_\alpha) + 2\pi R_\beta^2(1-\cos\theta_\beta) \\
V_1 &= \frac{\pi}{3}R_\alpha^3(2+\cos\theta_\alpha)(1-\cos\theta_\alpha)^2 \\
V_2 &= \frac{\pi}{3}R_\beta^3(2+\cos\theta_\beta)(1-\cos\theta_\beta)^2 \\
V &= V_1 + V_2 \\
A_r &= A^{3/2}/(6V\sqrt{\pi}) \\
R_\alpha &= R_\beta \sin\theta_\beta/\sin\theta_\alpha
\end{aligned}
\tag{S1}
$$

including vesicle area, droplet volumes and geometric constraint between droplet radii and angles. We then varied the excess area A_r and found the corresponding vesicle shapes by solving for $R_\alpha, R_\beta, \theta_\alpha, \theta_\beta$. Setting interfacial surface and membrane tensions $\Sigma_{\alpha\beta}, \Sigma_{\alpha\gamma}, \Sigma_{\beta\gamma}$ obtained from simulations, the free energy F_2 was then obtained as a function of excess area A_r assuming a zero line tension $\lambda = 0$, see the dashed line in Figure S3C.

The shape of the equilibrated vesicle, however, can be alternatively obtained by fitting spherical caps to the simulated vesicle as shown in Figure S2A. Spherical caps were fitted to the lipid tail groups by minimizing the least square distance subject to constraints on volumes V_1 and V_2 of the two liquid phases, see purple and red curves in Figure S3A, resulting in $A_r = 1.08$ for the vesicle shown there. For zero line tensions $\lambda = 0$, the free energy minimum did not occur at the obtained excess area $A_r = 1.08$. We thus changed the line tension λ to shift the free energy curve. The particular line tensions λ for which the free energy minimum occurred at the specific A_r found from simulation, $A_r = 1.08$ here, gave the line tension for that vesicle. As shown in the solid curve in Figure S2C, we found $\lambda = -2.6\ k_BT$ for this particular vesicle with given set of Δ and $\Sigma_{\alpha\beta}$, i.e. the separation propensity p.

The same procedure was repeated to find line tensions for various vesicles with different membrane asymmetries and separation propensities as listed in Tables S1-S3. Like interfacial tension $\Sigma_{\alpha\beta}$, the line tension λ also depends on the separation propensity p between the liquids as shown in Figure 1D.

S2. The free energy

The free energies of the two- and three-compartment vesicles, given in Equations 4 and 5, were computed by fitting spherical caps to membrane segments which results in bending

energies, and vesicle geometric parameters defined for the two-compartment vesicle n = 2 as

$$
\begin{aligned}
E_{b2} &= 2\kappa \frac{A_{\alpha\gamma}}{R_\alpha^2} + 2\kappa \frac{A_{\beta\gamma}}{R_\beta^2} \\
A_{\alpha\beta} &= \pi R_{co}^2 \\
A_{i\gamma} &= 2\pi R_i^2 (1 - \cos\theta_i) \quad (i = \alpha, \beta) \\
l_2 &= 2\pi R_{co}
\end{aligned}
\tag{S2}
$$

and for the three-compartment vesicle n = 3 as

$$
\begin{aligned}
E_{b3} &= 2\kappa \frac{A_{\alpha\gamma}}{R_\alpha^2} + 2\kappa \frac{A_{\beta\gamma L}}{R_{\beta L}^2} + 2\kappa \frac{A_{\beta\gamma R}}{R_{\beta R}^2} \\
A_{\alpha\beta L} &= \pi R_{coL}^2 \\
A_{\alpha\beta R} &= \pi R_{coR}^2 \\
A_{\alpha\gamma} &= 2\pi R_\alpha^2 (1 - \cos\theta_\alpha) \\
A_{\beta\gamma i} &= 2\pi R_{\beta i}^2 (1 - \cos\theta_{\beta i}) \qquad\qquad (i = L, R) \\
l_3 &= 2\pi R_{coL} + 2\pi R_{coR}
\end{aligned}
\tag{S3}
$$

where subscripts R and L, used in F_3, denote the right and left droplets of the liquid phase β and κ is the membrane bending rigidity. Table S4 lists the vesicle parameters for the specific vesicles shown in the right panels of Figure 1A,B.

Supplementary Figures

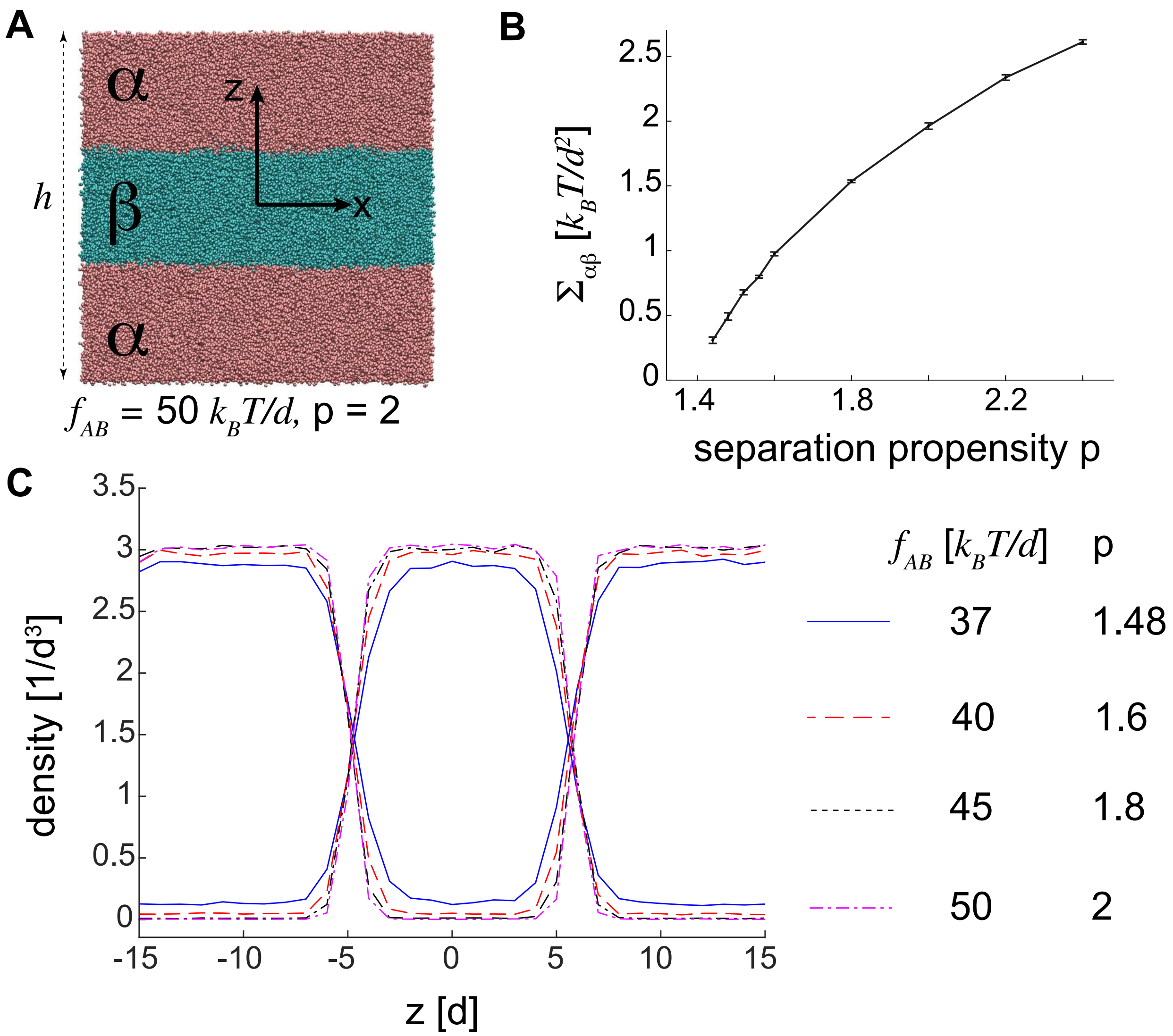

Figure S1. (A) Liquid phases α and β (pink and cyan beads, respectively) placed in a periodic simulation box to calculate the interfacial tensions $\Sigma_{\alpha\beta}$ at the interface of two liquid phases for $p = 2$. (B) Interfacial tension $\Sigma_{\alpha\beta}$ as a function of p. The two liquids show distinct separation for $p = 1.48$ as seen from density profiles along z axis in panel (C).

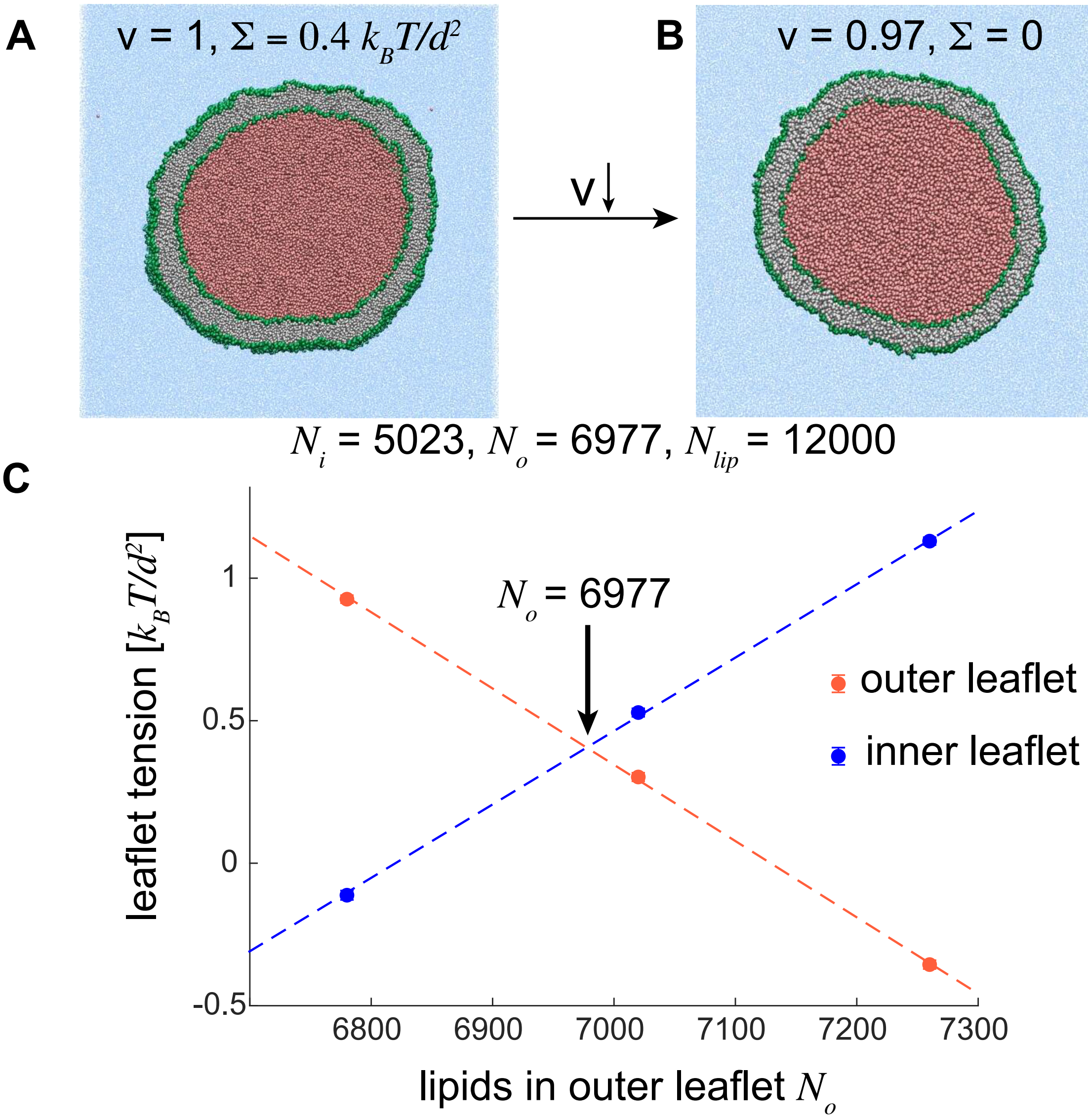

Figure S2. (A) An equilibrated reference nanovesicle (cross section) with $\Delta = \Delta_r = 0.16$ and a single internal liquid phases α (pink beads) separated from the surrounding liquid phase γ (blue beads), initially at volume $v = 1$. The vesicle, with a total number $N_{lip} = 12000$ of lipids, has a unique combination of lipid numbers $N_i = 5023$ and $N_o = 6977$ in its inner and outer leaflets, respectively, resulting in equal leaflet tensions, $\Sigma_i = \Sigma_o \approx 0.41\ k_BT/d^2$. (B) A slight decrease in the vesicle volume to $v = 0.97$ resulted in a tensionless vesicle with $\Sigma = 0$. (C) Membrane tensions in the inner and the outer leaflets of the vesicle for different combinations of N_i and N_o. The intersection of the two fitted lines gives the lipid composition of the reference vesicle $N_o = 6977$ and $N_i = 5023$ and thus the corresponding value of $\Delta = \Delta_r = 0.16$.

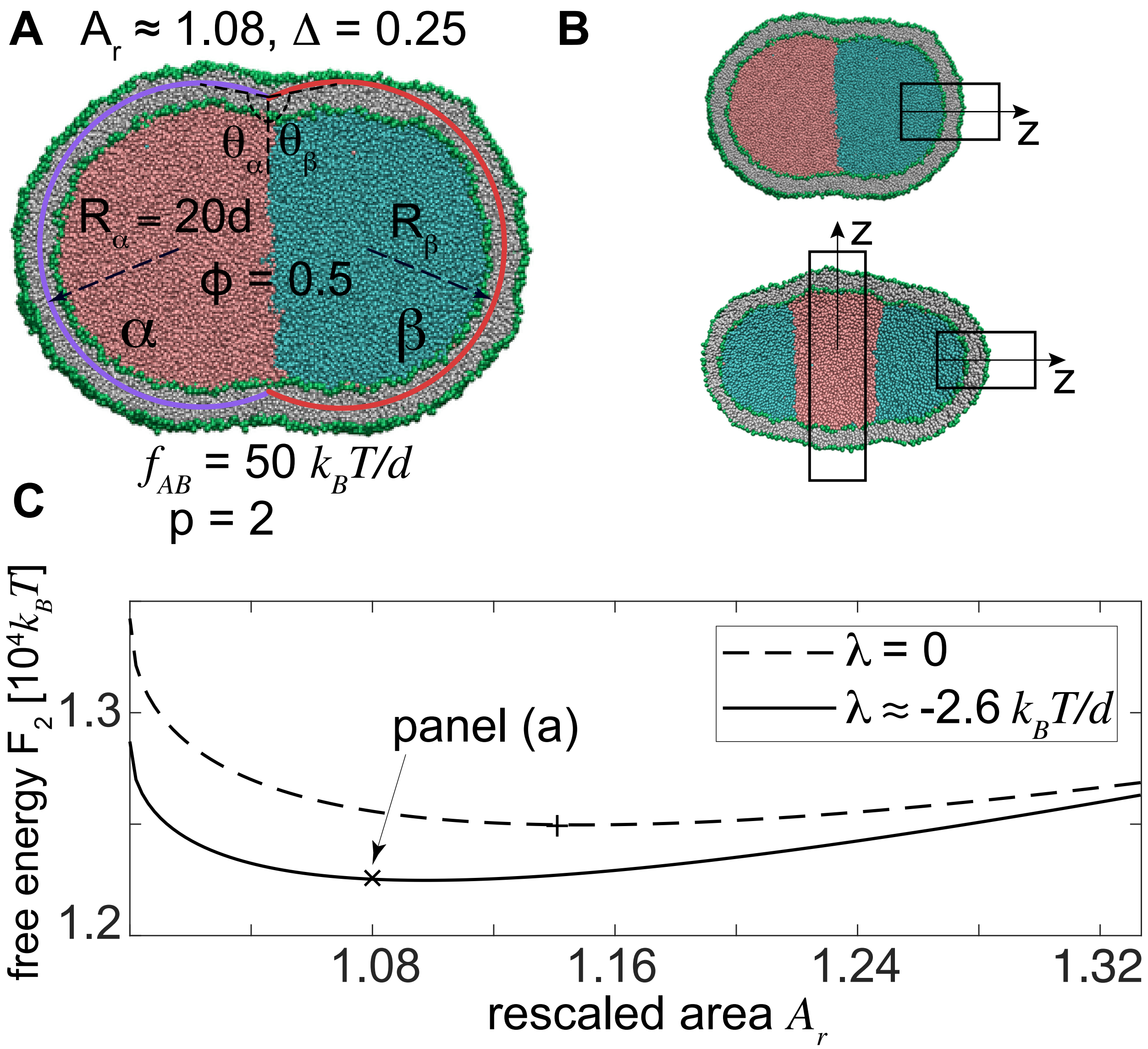

Figure S3. (A) An equilibrated stable two-compartment nanovesicle with minimum free energy composed of two separated liquid phases α and β (pink and cyan beads, respectively) with $\phi = 0.5$ as shown in Figure 1A. During equilibration, the area slightly increased at constant volume leading to an excess area A_r as listed in Tables S1 to S3, obtained by fitting two spherical caps with radii R_α and R_β (violet and red lines) with corresponding angles θ_α and θ_β to the simulations results. The spherical caps are fitted to the lipid tail groups using least square distance minimization. (B) The schematic diagram showing rectangular Cartesian boxes used to calculate the stress profile in the membrane along z direction, the area under which gives the membrane tension. (C) For the line tension $\lambda = 0$, the minimum energy occurred at the rescaled area $A_r = 1.14$ (cross) while simulated vesicle had an area $A_r = 1.08$ (star). A line tension λ (-2.6 k_BT/d) then shifted the minimum to the obtained $A_r = 1.08$ as shown in the lower curve, leading to the line tension in the nanovesicle in panel (A).

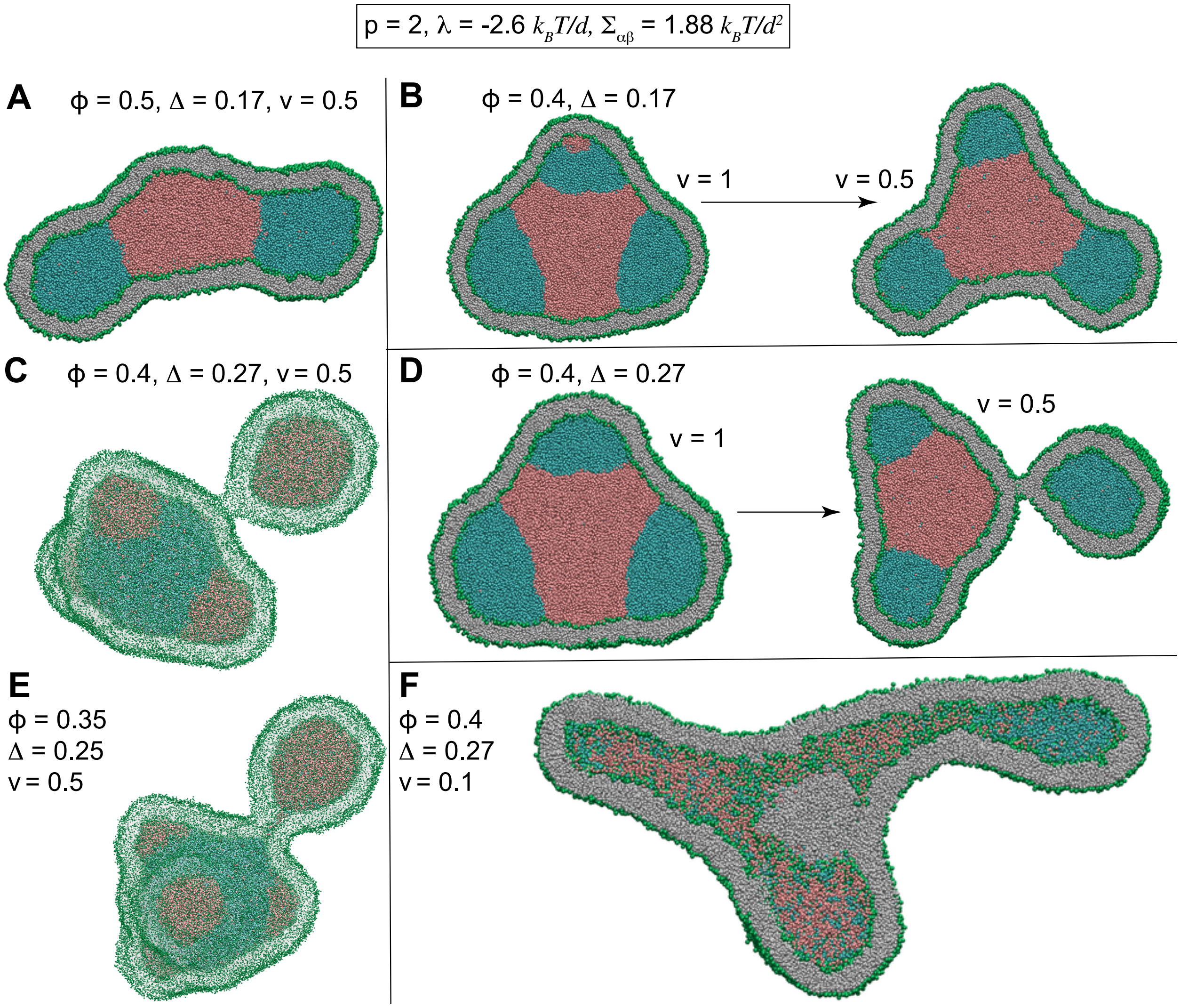

Figure S4. 3D view (C,E) and cross sections (A,B,D,F) of stable and meta-stable compartmentalized nanovesicles with different values of ϕ, v and Δ for $p = 2$ corresponding to $\lambda = -2.6\ k_BT/d$. (A) A tubular meta-stable nanovesicle at small $\Delta = 0.17$ and intermediate volume $v = 0.5$, partially stabilized due to negative line tension contribution. (B) A meta-stable four-compartment nanovesicle with $\Delta = 0.17$ transformed into a meta-stable starfish structure, with a long lifetime, upon volume reduction from $v = 1$ to $v = 0.5$. (C,E) Multi-compartment nanovesicles with n = 5 and 6 compartments at high membrane asymmetries $\Delta = 0.27$ and 0.57 with relatively small $\phi = 0.35$ and 0.4. (D) A meta-stable four-compartment nanovesicle with large $\Delta = 0.27$ transformed into a tube attached to a budded vesicle upon volume reduction from $v = 1$ to $v = 0.5$. (E) A meta-stable six-compartment nanovesicle. (F) A branched tubule for high $\Delta = 0.27$.

Supplementary Tables

Table S1: Vesicle parameters for a stable vesicle with two compartments formed by two initially separated liquid phases α and β (Figure 1A) with $\phi = 0.5$ and $p = 1.6$, corresponding to $\lambda = -0.77\ k_BT/d$ and $\Sigma_{\alpha\beta} = 0.87\ k_BT/d^2$. Corresponding vesicles are shown in the second row of Figure 4 with different membrane asymmetries $0.13 \leq \Delta \leq 0.25$.

$p = 1.6$

	$\Delta = 0.13$	$\Delta = 0.17$	$\Delta = 0.21$	$\Delta = 0.25$	units
N_i	5220	4980	4740	4500	
N_o	6780	7020	7260	7500	
N_w	116000	116000	118000	120000	
R_α	21.4 ± 0.7	21.4 ± 0.7	21.4 ± 0.8	21.4 ± 0.8	d
R_β	20.7 ± 0.7	20.7 ± 0.7	20.6 ± 0.8	20.6 ± 0.8	d
R_{co}	19.3 ± 0.3	19.3 ± 0.2	19.2 ± 0.3	19.1 ± 0.2	d
A_r	1.05 ± 0.004	1.06 ± 0.003	1.1 ± 0.004	1.06 ± 0.003	d^2
θ_α	115.2 ± 1.4	115.3 ± 1	116.1 ± 1.6	116.9 ± 0.6	degrees
θ_β	110.9 ± 1.1	111 ± 1	111.3 ± 0.8	112 ± 1	degrees
$\Sigma_{\alpha\gamma}$	0.8 ± 0.06	0.7 ± 0.2	0.8 ± 0.1	0.7 ± 0.1	k_BT/d^2
$\Sigma_{\beta\gamma}$	0.76 ± 0.06	0.8 ± 0.06	0.8 ± 0.1	0.7 ± 0.07	k_BT/d^2
$\Sigma_{\alpha\beta}$	0.87 ± 0.1	0.86 ± 0.2	0.88 ± 0.1	0.9 ± 0.04	k_BT/d^2
λ	-0.77 ± 0.4	-0.77 ± 0.4	-0.77 ± 0.4	-0.77 ± 0.4	k_BT/d

Table S2: Vesicle parameters for a stable vesicle with two compartments formed by two initially separated liquid phases α and β (Figure 1A) with $\phi = 0.5$ and $p = 1.8$, corresponding to $\lambda = -1.8\ k_BT/d$ and $\Sigma_{\alpha\beta} = 1.4\ k_BT/d^2$. Corresponding vesicles are shown in the third row of Figure 4 with different membrane asymmetries $0.13 \leq \Delta \leq 0.25$.

$p = 1.8$

	$\Delta = 0.13$	$\Delta = 0.17$	$\Delta = 0.21$	$\Delta = 0.25$	units
N_i	5220	4980	4740	4500	
N_o	6780	7020	7260	7500	
N_w	116000	116000	118000	120000	
R_α	20.8 ± 0.6	20.9 ± 0.7	20.9 ± 0.6	20.9 ± 0.6	d
R_β	20.3 ± 0.6	20.2 ± 0.7	20.3 ± 0.6	20.3 ± 0.6	d
R_{co}	18.1 ± 0.2	18 ± 0.3	18 ± 0.2	18 ± 0.3	d
A_r	1.1 ± 0.003	1.1 ± 0.004	1.1 ± 0.004	1.1 ± 0.005	d^2
θ_α	119.7 ± 1.2	120.4 ± 1.2	120.2 ± 1	120.8 ± 1.5	degrees
θ_β	116.8 ± 0.5	116.8 ± 1	117.2 ± 0.8	117.9 ± 1.2	degrees
$\Sigma_{\alpha\gamma}$	1.1 ± 0.1	1.1 ± 0.1	1 ± 0.1	1.1 ± 0.1	k_BT/d^2
$\Sigma_{\beta\gamma}$	1.1 ± 0.1	1.1 ± 0.1	1.2 ± 0.1	1.1 ± 0.1	k_BT/d^2
$\Sigma_{\alpha\beta}$	1.4 ± 0.1	1.4 ± 0.1	1.4 ± 0.2	1.4 ± 0.04	k_BT/d^2
λ	-1.8 ± 0.4	-1.8 ± 0.4	-1.8 ± 0.4	-1.8 ± 0.4	k_BT/d

Table S3: Vesicle parameters for a stable vesicle with two compartments formed by two initially separated liquid phases α and β (Figure 1A) with $\phi = 0.5$ and $p = 2$, corresponding to $\lambda = -2.6\ k_BT/d$ and $\Sigma_{\alpha\beta} = 1.88\ k_BT/d^2$. Corresponding vesicles are shown in the last row of Figure 4 with different membrane asymmetries $0.13 \leq \Delta \leq 0.25$.

$p = 2$

	$\Delta = 0.13$	$\Delta = 0.17$	$\Delta = 0.21$	$\Delta = 0.25$	units
N_i	5220	4980	4740	4500	
N_o	6780	7020	7260	7500	
N_w	116000	116000	118000	120000	
R_α	20.6 ± 0.5	20.7 ± 0.6	20.7 ± 0.6	20.8 ± 0.7	d
R_β	20.1 ± 0.5	20 ± 0.6	20.1 ± 0.6	20.1 ± 0.7	d
R_{co}	17.5 ± 0.2	17.4 ± 0.2	17.5 ± 0.2	17.4 ± 0.2	d
A_r	1.1 ± 0.003	1.1 ± 0.003	1.1 ± 0.003	1.1 ± 0.003	d^2
θ_α	121.9 ± 0.7	122.5 ± 0.8	122.2 ± 0.8	123 ± 0.9	degrees
θ_β	119.4 ± 0.6	119.6 ± 0.6	119.5 ± 0.7	120.1 ± 0.6	degrees
$\Sigma_{\alpha\gamma}$	1.35 ± 0.1	1.3 ± 0.05	1.3 ± 0.1	1.3 ± 0.1	k_BT/d^2
$\Sigma_{\beta\gamma}$	1.3 ± 0.1	1.3 ± 0.1	1.3 ± 0.1	1.35 ± 0.1	k_BT/d^2
$\Sigma_{\alpha\beta}$	1.88 ± 0.1	1.85 ± 0.1	1.88 ± 0.1	1.88 ± 0.1	k_BT/d^2
λ	-2.6 ± 0.4	-2.6 ± 0.4	-2.6 ± 0.4	-2.6 ± 0.4	k_BT/d

Table S4: Vesicle parameters for two/three-compartment nanovesicles with $\Delta = 0.17$ and two/three compartments formed by two/three initially separated liquid phases α and β (Figure 1A,B) with $\phi = 0.5$ and $p = 2$, corresponding to $\lambda = -2.6\ k_BT/d$ and $\Sigma_{\alpha\beta} = 1.88\ k_BT/d^2$, as shown in the line tension graph in Figure 1D and listed in Table 3.

$p = 2$, $\Delta = 0.17$

n = 2			n = 3		
θ_α	122.5 ± 0.8	degrees	θ_α	124 ± 0.5	degrees
θ_β	119.6 ± 0.6	degrees	$\theta_{\beta L}$	104.5 ± 1	degrees
R_α	20.7 ± 0.6	d	$\theta_{\beta R}$	104.5 ± 0.8	degrees
R_β	20 ± 0.6	d	R_α	20.7 ± 0.1	d
R_{co}	17.4 ± 0.2	d	$R_{\beta L}$	17.6 ± 0.1	d
$\Sigma_{\alpha\gamma}$	1.3 ± 0.04	k_BT/d^2	$R_{\beta R}$	18 ± 0.1	d
$\Sigma_{\beta\gamma}$	1.3 ± 0.1	k_BT/d^2	R_{coL}	17.2 ± 0.1	d
			R_{coR}	17.4 ± 0.1	d
			$\Sigma_{\alpha\gamma}$	1.7 ± 0.1	k_BT/d^2
			$\Sigma_{\beta\gamma L}$	1.6 ± 0.1	k_BT/d^2
			$\Sigma_{\beta\gamma R}$	1.6 ± 0.1	k_BT/d^2

Supplementary Videos

SV1:

Video showing the evolution of a stable vesicle with $\Delta = 0.17$ and two separated liquid phases α and β (pink and cyan beads, respectively) with $\phi = 0.5$ whose volume was reduced from $v = 1$ to $v = 0.1$ in 90μs as the vesicle transformed into a sheet morphology. The two liquid phases completely mixed with $p = 1.4$ for which the line and interfacial tensions are not defined in the absence of the interfacial surface and thus do not contribute to the vesicle free energy.

SV2:

Video showing the evolution of a stable vesicle with two separated liquid phases α and β (pink and cyan beads, respectively) with $\phi = 0.5$ whose volume was reduced from $v = 1$ to $v = 0.1$ in 90μs as the vesicle transformed into a cup with $\Delta = 0.13$. The two initially-separated liquid phases remained separated with $p = 1.6$ corresponding to $\lambda = -0.77\ k_BT/d$ and $\Sigma_{\alpha\beta} = 0.87\ k_BT/d^2$.

SV3:

Video showing the evolution of a stable vesicle with two separated liquid phases α and β (pink and cyan beads, respectively) with $\phi = 0.5$ whose volume was reduced from $v = 1$ to $v = 0.1$ in 90μs as the vesicle transformed, via an intermediate dumbbell, into a tubule with $\Delta = 0.25$. The two initially-separated liquid phases remained separated with $p = 1.6$ corresponding to $\lambda = -0.77\ k_BT/d$ and $\Sigma_{\alpha\beta} = 0.87\ k_BT/d^2$.

SV4:

Video showing the phase-separation of two initially randomly mixed liquid phases α and β (pink and cyan beads, respectively) with $\phi = 0.5$, actively compartmentalizing a spherical nanovesicle with $\Delta = 0.17$. The two liquid phases with $p = 2$ phase-separated rapidly in about 5 μs.

SV5:

Video showing the morphological transformations of a three-compartment meta-stable nanovesicle with $\phi = 0.5$ and $\Delta = 0.17$, with fixed volume $v = 1$ for about 100 μs. Despite the instability of the three-compartment nanovesicle, the two liquid nanodroplets of the liquid phase β (cyan beads) did not coalesce even in a relatively long simulation time, presumably due to the stabilizing role of large negative line tension $\lambda = -2.6\ k_BT/d$ for $p = 2$, which disfavored droplet coalescence. Different terms of the vesicle free energy F_3 are given in Equation 3.

SV6:

Video showing the morphological transformations of a four-compartment meta-stable nanovesicle with $\phi = 0.4$ and $\Delta = 0.21$, whose volume was slowly reduced from $v = 1$ to $v = 0.1$ in about 90μs during which the nanovesicle maintained its branched tubular structure while evolving into narrower tubular branches. Line and interfacial tensions were $\lambda = -2.6\ k_BT/d$ and $\Sigma_{\alpha\beta} = 1.88\ k_BT/d^2$ for $p = 2$.

SV7:

Video showing the morphological transformations of a three-compartment meta-stable nanovesicle with $\phi = 0.4$ and $\Delta = 0.27$, whose volume was slowly reduced from $v = 1$ to $v = 0.5$ in about 50 μs during which the nanovesicle transformed into a budded vesicle connected to a

two-compartment smaller nanovesicle by a narrow neck. Line and interfacial tensions were $\lambda = -2.6\ k_BT/d$ and $\Sigma_{\alpha\beta} = 1.88\ k_BT/d^2$ for $p = 2$.

SV8:

Video showing the morphological transformations of a three-compartment meta-stable nanovesicle with $\phi = 0.4$ and $\Delta = 0.27$, whose volume was slowly reduced from $v = 1$ to $v = 0.1$ in about 90 μs during which the nanovesicle transformed into a long tubular nanovesicle via an intermediate budded vesicle connected to a two-compartment smaller nanovesicle by a narrow neck. Line and interfacial tensions at the liquid interfaces were $\lambda = -2.6\ k_BT/d$ and $\Sigma_{\alpha\beta} = 1.88\ k_BT/d^2$ for $p = 2$.
